# The therapeutic potential of icariin in the intervention of bone and joint diseases through multiple pathways: a narrative review

**DOI:** 10.3389/fphar.2025.1606912

**Published:** 2025-11-07

**Authors:** Jiayi Luo, Lijiao Wu, Yunying Xu, Jingqi Zhang, Xinyi Zhao, Xiangjin Wang

**Affiliations:** 1 Shanghai Shuguang Hospital affiliated to Shanghai University of Traditional Chinese Medicine, Shanghai, China; 2 Hospital of Chengdu University of Traditional Chinese Medicine, Chengdu, China; 3 College of Medicine and Life Sciences, Chengdu University of Traditional Chinese Medicine, Chengdu, China; 4 College of lntegrated Traditional Chinese and Western Medicine, Hunan University of Chinese Medicine, Changsha, China

**Keywords:** icariin, natural flavonoids, Osteometabolic balance, inflammation, oxidativestress, mechanisms

## Abstract

The prevalence of bone and joint diseases is projected to increase owing to rapidly aging populations, sedentary lifestyles, and unhealthy diets. This poses a significant challenge for the global healthcare system. In recent years, natural herbal medicines have been used to treat various types of orthopedic diseases, opening new frontiers in new drug research. Epimedium is a traditional Chinese herb with a long history of medicinal use. It is commonly used to treat osteoporosis, joint disorders, cardiovascular diseases, sexual dysfunction and aging. The primary active component of Epimedium is icariin (ICA), an isoprenylated flavonoid. Recent studies have demonstrated its significant positive effects on bone metabolism and remodeling, including promoting osteoblast proliferation and mineralization, reducing osteoclast activity, and inhibiting inflammation and oxidative stress. Thus, ICA represents a potential compound for treating bone and joint diseases. However, the specific mechanisms underlying these effects have not yet been fully elucidated. This paper focuses on the latest advances in the use of ICA for the treatment of skeletal and joint diseases, covering a range of conditions including osteoporosis, osteoarthritis, rheumatoid arthritis, intervertebral disc degeneration and fractures. Building on this, we have systematically integrated its multi-target pharmacological mechanism network for the first time, elucidating its multi-pathway synergistic effects mediated by regulating the balance of the bone microenvironment. In summary, as a multi-target natural compound, ICA demonstrates significant translational medicine potential in the comprehensive treatment of bone and joint diseases, providing a critical theoretical foundation for the development of novel therapeutic strategies.

## 1 Introduction

Epimedium, the largest herbaceous genus in the Berberidaceae family, comprises approximately 62 species (Zhuang et al., 2023). These plants have a discontinuous distribution from Algeria in North Africa to East Asia, where the majority of the species (approximately 52) have undergone continuous evolution (Wang J. et al., 2022; Zhang et al., 2022b). Certain species of Epimedium have been used in traditional Asian medicine and have been shown to have significant therapeutic activity (Liang et al., 2025). Epimedium was first recorded in Shennong Ben Cao Jing and is recognized as a “medium” herb in the most famous Chinese medical text, Ben Cao Gang Mu (Zhang et al., 2022a).

Epimedium dispels wind and disperses cold, tonifies the kidneys, and strengthens tendons (Ma H. et al., 2011). Traditional medicine posits that tonifying kidney yang can nourish kidney essence, thereby promoting bone marrow production and nourishing bones to strengthen tendons and bones, as well as alleviate joint stiffness and pain (e.g., rheumatic pain and impaired joint flexion/extension). Additionally, according to traditional Chinese medical theory, “obstruction of meridians by wind, cold, and dampness is the root cause of arthralgia.” Possessing wind-cold-dispelling properties, Epimedium can eliminate wind-cold-dampness pathogens invading the tendons and bones, dredge qi and blood, and thus synergize with its effects of tonifying the kidney and strengthening the tendons. Owing to its therapeutic effects on the bones and kidneys, it is used as a traditional medicine for treating osteoporosis, joint disorders, cardiovascular disorders, sexual dysfunction, as well as slowing aging in many countries and regions (Guo et al., 2022; Wang et al., 2025). Therefore, Epimedium has great potential for research and development.

Previous studies have identified more than 260 compounds in epimedium, including flavonoids, lignans, violacein, phenolic glycosides, and other compounds representing various categories of secondary metabolites (Wu et al., 2003). The most well-known and phytochemically characteristic of these compounds are flavonoids, with the most prominent components being isoprenylated flavonoid glycosides (Lin et al., 2023). As the flavonoid component with the highest content and most extensive research, icariin (ICA) has been identified as the primary material basis mediating the core pharmacological effects of Epimedium crude extracts, including anti-inflammatory activity, promotion of bone formation, and inhibition of bone resorption (Ma H. et al., 2011). In addition, the Chinese Pharmacopoeia also designates ICA as a chemical marker for the quality control of Epimedium (Zhou et al., 2015). ICA has been shown to possess several pharmacological effects, including osteoprotective, anti-osteoporotic, anti-inflammatory and antioxidant capabilities, as well as enhancing cardiovascular function, neuroprotection, and regulating the immune system (Ming et al., 2013; Buhrmann et al., 2020; He et al., 2020; Yan et al., 2023). Intensive research on the effects of ICA on bone damage has attracted attention to its potential as a therapeutic agent for the treatment of bone and joint diseases.

Bone and joint diseases are a major societal burden affecting people worldwide (Imagama et al., 2020). Generally, they arise from abnormal metabolism and cell death in the skeletal system, including osteoblasts, osteoclasts, chondrocytes, and bone marrow mesenchymal stem cells (BMMSCs) (Zeng et al., 2022). A precise balance between bone resorption and formation is crucial for bone homeostasis (Li et al., 2022). Imbalance in bone homeostasis is one of the major causes of the development of certain bone diseases, including osteoporosis (OP), osteoarthritis (OA), rheumatoid arthritis (RA), intervertebral disc degeneration (IVDD), and delayed healing/nonunion fractures (Wang S. et al., 2020; Zeng et al., 2022). In addition, trauma, substance abuse, cartilage degeneration, hormonal imbalance, and aging are potential risk factors for the development of bone disease (Cao et al., 2017; Luo et al., 2018). Currently, the standard treatments for bone diseases include surgery-based revascularization, joint replacement, oral pharmacological agents, rehabilitation in non-surgical programs, and alternative therapies (McCormick et al., 2014; Tonomura et al., 2020). Although these strategies are frequently updated with advances in medicine and play a crucial role in disease prevention and treatment, they have some drawbacks, such as irreversible complications, high costs, limitations in long-term use, and side effects (Ruderman, 2012; Sugiyama et al., 2015). Side effects have been reported with long-term use of anti-osteoporotic drugs, such as bisphosphonates, which can cause upper gastrointestinal bleeding, hypersensitivity reactions, hypocalcemia and myalgia (Papapetrou, 2009); and use of teriparatide for more than 2 years may increase the risk of developing osteosarcoma (Ma et al., 2004). In recent years, natural Chinese medicines have been used to treat various orthopedic conditions owing to their excellent efficacy, high safety and few side effects (Mukwaya et al., 2014). ICA, a natural compound extracted from traditional Chinese medicine (TCM), plays an important role in the treatment of bone and joint diseases. ICA possesses osteoinductive potential for bone tissue engineering and contributes to the regulation of various signaling pathways, including those involved in anti-osteoporosis, osteogenesis, anti-fracture, chondrogenesis, angiogenesis and anti-inflammation (Zhang et al., 2014). Numerous animal studies have confirmed the osteoprotective effects of ICA *in vitro* and *in vivo*. One study found that ICA promoted osteogenic differentiation *in vitro* and alleviated osteoporosis *in vivo* by inhibiting the Notch signaling pathway (Xu Y. et al., 2019). Another *in vivo* study showed that ICA treatment reduced articular cartilage damage in OA rats by inhibiting cartilage extracellular matrix (ECM) degradation and chondrocyte iron death (Xiao et al., 2024). Angiogenesis also plays an important role in bone regeneration, and it has been reported that ICA can enhance angiogenesis through endothelial cell migration, proliferation and renal therapy *in vivo* (Chung et al., 2008). Additionally, ICA can be stabilized for local release using biomaterials, making it a promising drug candidate for the treatment of bone and joint disorders (Zhao et al., 2010).

Although existing studies have confirmed the multifaceted roles of ICA in skeletal and joint diseases, its mechanisms of action remain fragmented: on the one hand, the signaling pathways regulated by ICA in different diseases lack cross-disease systemic associations; on the other hand, the interactions between emerging mechanisms such as oxidative stress and autophagy and classical osteogenic/osteoclastic pathways remain unclear, and research linking drug toxicity to clinical translation is relatively weak. Based on this, this review systematically integrates the cross-disease regulatory networks of ICA in various diseases such as OP and OA, breaking through the limitations of single-disease mechanism studies; it focuses on elucidating the interactions between emerging mechanisms such as oxidative stress and autophagy and classical signaling pathways; simultaneously, it offers forward-looking insights into drug toxicity assessment and clinical translation strategies, providing a more comprehensive theoretical foundation for the full-chain development of ICA from basic research to clinical application—an area previously under-explored in systematic research.

## 2 Epimedium and its active ingredient–icariin

Herbal Epimedium is prepared from the dried leaves of the Epimedium medicinal plant. Epimedium has been used in traditional Chinese medicine for over 2000 years, and is commonly used to treat bone fractures, joint ailments, chronic diseases and aging (Gao et al., 2022). The primary pharmacologically active compounds in Epimedium are isopentenyl flavonol glycosides, with various sugar groups at the 3-OH and 7-OH positions. Among these compounds, epimedin A, B, and C exhibit certain biological activities (Shen et al., 2007a). However, ICA is currently the most extensively studied and mechanistically well-defined core active monomer, which can be extracted from the dried stems and leaves of E. sagittatum Maxim., E. pubescens Maxim., E. brevicornu Maxim., E. koreanum Nakai, and E. wushanense T. S. Ying (Liu et al., 2023).

**FIGURE 1:**
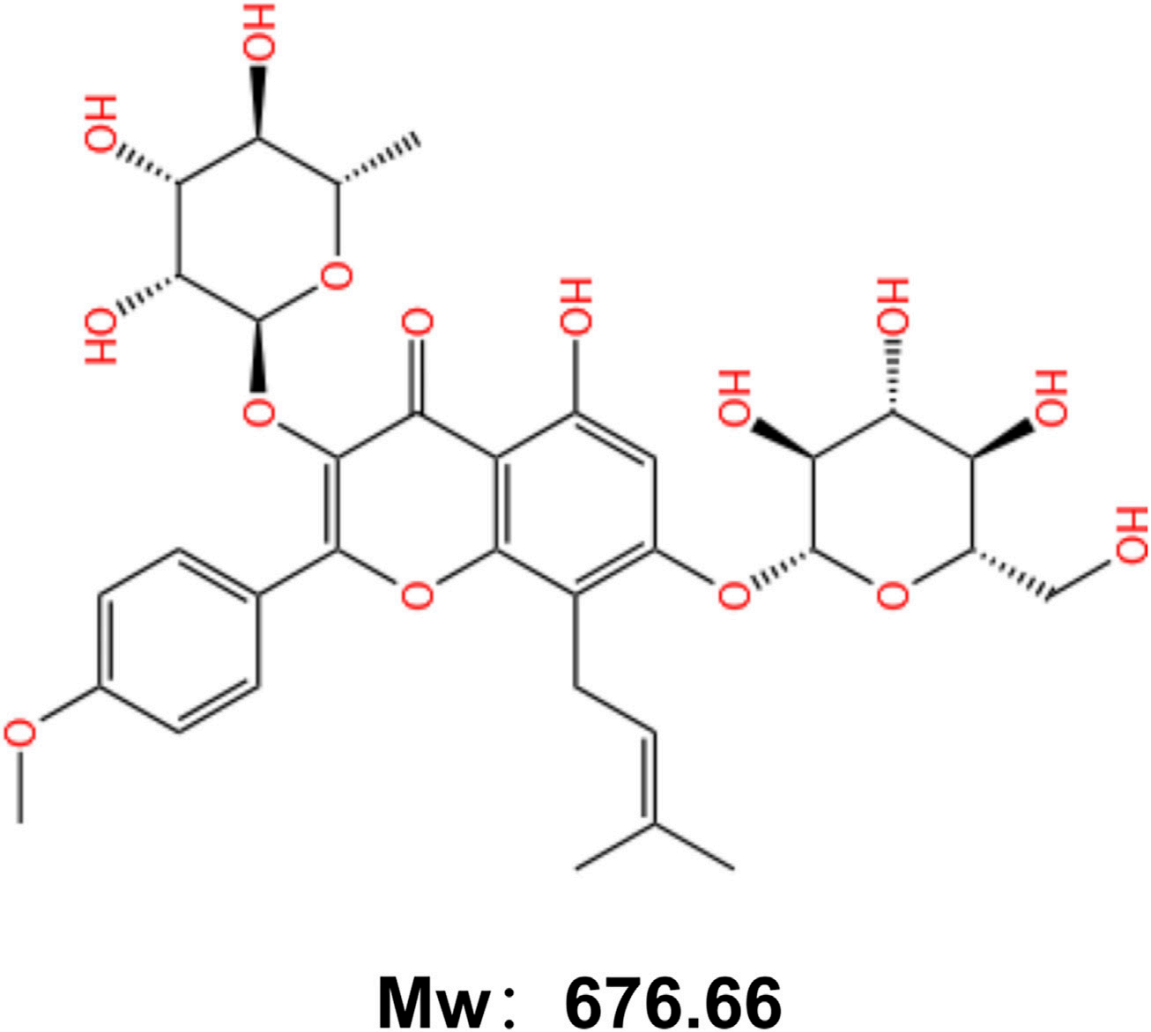
Chemical structure of Icariin.

ICA (C33H40O15, molecular weight: 676.66) (Yang et al., 2019) is an isoprenyl flavonoid that appears as a yellowish powder (Figure 1). It is composed of a glucose group at C-3, a methoxyl group at C-4, a prenyl group at the C-8 position and a rhamnosyl group at C-7 (He et al., 2020). This structure endows ICA with diverse pharmacological activities. Recent research has focused on its bone-protective, anti-inflammatory, anti-cancer, and immunomodulatory effects (Li Y. et al., 2024), demonstrating significant potential particularly in the treatment of bone and joint diseases.

Currently, multiple studies have been conducted to provide a global perspective on the pharmacokinetics of ICA. The pharmacokinetic properties of ICA are closely linked to the method of administration. Xu et al. investigated the absorption behavior of ICA in rats following oral or intramuscular injection and discovered that the half-life of orally administered ICA was 3.149–2.364 h (Xu et al., 2017). Intravenously administered ICA exhibited a rapid decrease in blood concentration, with a half-life of 0.562–0.200 h. They also investigated the metabolic pattern of ICA and found that plasma and tissue concentrations of ICA remained very low at all collection sites, suggesting that epimedium glycosides are poorly absorbed after oral administration. In addition, the concentrations were much higher in the liver, lung and reproductive organs than in other tissues, suggesting that the liver, lung and reproductive organs are the main targets of ICA in rats. Ye et al. found that ICA is difficult to cross the blood-brain barrier (Lika et al., 1999), but peripheral administration of ICA has also been reported to have significant central nervous system pharmacological activity (Jin et al., 2014; Li F. et al., 2015). In addition, the distribution of ICA varied with concentration and sex. For instance, at moderate concentrations, ICA is present in the testicles of male rats and at higher concentrations in the uterus and ovaries of female rats (Xu et al., 2017). Further, it has been found that ICA metabolizes to Epimedium and desmethyl Epimedium in humans (Shen et al., 2007b), whereas it is only metabolized to desmethyl Epimedium in SD rats, suggesting species-dependent metabolism (Wong et al., 2009). Therefore, a more thorough metabolic profile of ICA in humans needs to be established. For orally administered drugs, the most important pharmacokinetic property is their bioavailability (Tan et al., 2021). Notably, like other flavonoids, ICA and its derivatives have low oral bioavailability (∼12%), which may be due to the low water solubility and membrane permeability of ICA and the slow dissolution rate in biological fluids (Jin et al., 2019). Several methods have been developed to improve the oral bioavailability of ICA. In the most successful study (Szabó et al., 2022), a 6.57-fold increase in bioavailability was achieved using phospholipid complex formation. In addition, the simultaneous application of several different techniques may result in a synergistic effect that exponentially increases the bioavailability of insoluble flavonoids. These cutting-edge studies open up the possibility of the therapeutic potential of ICA in bone and joint diseases.

**FIGURE 2:**
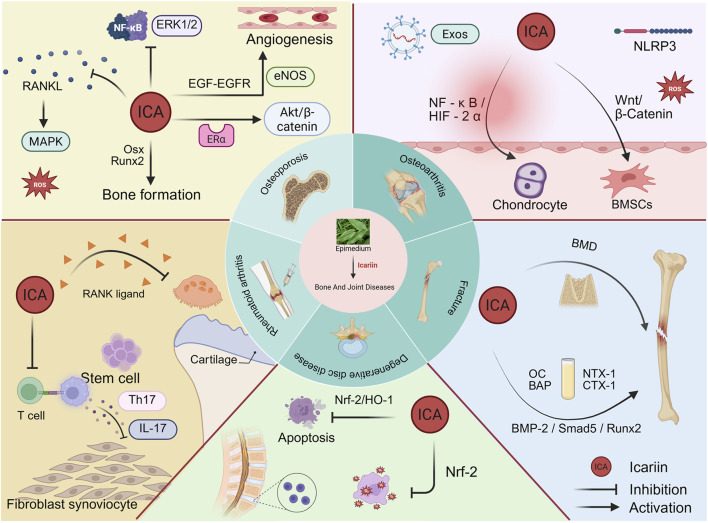
Therapeutic potential of Icariin in bone and joint disorders.

Positive advances in the pharmacological activities of ICA have been made in recent years in relation to the skeletal system, cancer, nervous system, immune system and cardiovascular system (Li C. et al., 2015). A series of studies have demonstrated multiple mechanisms through which ICA treats bone and joint diseases, including the induction of bone formation, inhibition of bone resorption and angiogenesis (Wang et al., 2018). For example, Wang et al. found that ICA and five other flavonoids enhance extracellular matrix mineralization, promote osteoblast proliferation and differentiation, and facilitate the osteogenic differentiation of bone marrow mesenchymal stem cells (BMSCs) (Wang et al., 2024). Additionally, Li et al. confirmed that icariin not only inhibits osteoclast formation but also suppresses their bone resorption capacity (Li F. et al., 2024). Although clinical trials remain limited, existing research has revealed that ICA can treat bone and joint diseases through multiple pathways. However, these molecular mechanisms require further comprehensive summarization (Figure 2).

## 3 Molecular mechanism of icariin in treating bone and joint diseases

### 3.1 Osteoporosis

Osteoporosis (OP) is a chronic metabolic bone disease characterized by reduced bone density and microarchitecture, resulting in increased bone fragility and fracture risk (Li et al., 2023). OP affects over 200 million people worldwide, making it one of the most prevalent diseases among the elderly (Jia et al., 2012; Ballane et al., 2017). OP causes over 8.9 million osteoporotic fractures annually worldwide (Böcker et al., 2022), resulting in a significant socioeconomic burden. The primary treatments for OP include humanized receptor activator of nuclear factor-κΒ ligand (RANKL) monoclonal antibodies, bisphosphonates, selective estrogen receptor modulators and calcitonin (Liu et al., 2020). However, these therapeutic strategies are associated with significant adverse effects, including jaw osteonecrosis, hypercalcemia, breast cancer and cardiovascular disease (Fleisch, 2003; Barrett-Connor et al., 2006). The pathogenesis of OP mainly includes the loss of bone homeostasis, with the maintenance of bone homeostasis dependent on the appropriate balance between osteoblasts differentiated from BMMSCs and osteoclasts produced by the fusion of mononuclear macrophages (Song et al., 2022). Various transcription factors, such as osterix (Osx), runt-related protein 2 (Runx2), Wingless and INT-1 signals have been shown to regulate osteoblast differentiation and function (Long, 2011). Similarly, osteoclast differentiation and function are regulated by factors, such as RANKL, macrophage colony-stimulating factor (M-CSF), cytokines and αVβ 3 integrins (Teitelbaum, 2000). Additionally, numerous loci related to OP that regulate bone homeostasis have been identified. These include the low-density lipoprotein receptor-related protein 5 (LRP5), estrogen receptor α (ESR1) and osteoprotegerin (OPG) (Richards et al., 2008).

In addition, ICA inhibits osteoclastogenesis to treat OP by regulating RANKL-induced signaling pathways. RANKL primarily controls the function and survival of mature osteoclasts (Takayanagi et al., 2002). It binds to its receptor RANK, which is expressed on the surface of osteoclast precursor cells, whereas OPG serves as a protective factor for inducing osteoclast differentiation (Nakashima et al., 2012). Xu et al. discovered that ICA inhibited RANKL-induced phosphorylation of three MAPKs in RAW264.7 cells, indicating that ICA exerts an inhibitory effect on osteoclast differentiation by regulating MAPK phosphorylation in mature osteoclasts (Xu Q. et al., 2019). ICA administration also significantly restored serum calcium levels and OPG/RANKL ratios in osteoblasts, acting as a bone preservative in simulating microgravity-induced bone loss (He J. et al., 2018).In addition, ICA inhibits osteoclast differentiation by downregulating TRAF6, and inhibiting the activation of ERK1/2 and nuclear factor-κB (NF-κB), thereby effectively inhibiting RANKL-induced expression of nuclear factor of activated T cell cytosolic 1 (NFATc1) and c-Fos transcription factors, which are key factors regulating osteoclast-specific genes (Kim et al., 2018).

The decreased bone mass and increased bone marrow adipose tissue in OP is closely related to the dynamic balance between the differentiation of BMMSCs into adipocytes and osteoblasts. Inhibition of adipogenic differentiation of BMMSCs and promotion of osteogenic differentiation are important indices to validate the treatment of OP (Justesen et al., 2001). Several studies have shown that the maturation and mineralization of osteoblasts and osteogenic differentiation of BMMSCs during bone formation can be improved and enhanced by ICA treatment (Huang et al., 2007). Mechanistically, ICA exerts these effects through activation of the Wnt/β-catenin pathway, which is attributed to the potent anti-apoptotic activity of ICA (Chen et al., 2016). In addition, Zhang et al. demonstrated that mouse MC3T3 - E1 and RAW264.7 cells could be co-cultured *in vitro*, and ICA treatment increased the osteogenic activity of these cells (Zhang et al., 2017). ICA also promotes bone formation by inducing the expression of Osx, Runx2, alkaline phosphatase (ALP), type I collagen, and other proosteoblast genes to promote bone formation (Ma H. P. et al., 2011). In addition, ICA has been shown to increase the expression of Runx2 and Bmp2, while reducing the expression of CCAAT/enhancer binding protein alpha (C/EBPa) and peroxisome proliferator-activated receptor gamma (Pparγ). This suggests that ICA stimulates osteoblast differentiation and mineralization and inhibits osteoblast fatty degeneration, thereby increasing the number of osteoblasts that differentiate into mature osteocytes (Zhang et al., 2016). Ye et al. found that ICA promotes the proliferation and osteogenic differentiation of rat adipose stem cells through the Rho A-TAZ pathway at an optimal concentration of 10–7 M (Ye et al., 2017).

Another way that ICA acts as an antiosteoporotic agent is by preventing estrogen deficiency. Estrogen is used clinically for bone conditions because it can regulate bone formation and resorption in bone homeostasis. Estrogen receptor α (Erα) mediates the activity of estrogen in safeguarding cortical bone mass, which is crucial for osteoblast differentiation (Manolagas et al., 2013). Decreased estrogen concentration in postmenopausal women often reduces the amount and function of ERα, potentially resulting in osteoporosis (Song et al., 2008). Zhao et al. reported that ICA activates the Akt/β-catenin pathway by upregulating ERα expression through phytoestrogen administration in de-ovulated rats (Zhao et al., 2019). This promotes osteoblast proliferation and differentiation, leading to improved locomotor skeletal muscle response. ICA also promotes transcription of the downstream osteogenic gene osteocalcin (Ocn) through STAT3, thereby preventing estrogen deficiency-induced alveolar bone loss (Xu et al., 2020). In an *in vivo* study, Xu et al. elucidated a novel mechanism by which ICA promotes osteogenic differentiation and bone formation by inhibiting the Notch signaling pathway (Xu Y. et al., 2019). In addition, ICA rapidly induces extracellular signaling to promote the activation of extracellular signal-regulated kinase (ERK) and c-Jun N-terminal kinase (JNK), which effectively promote osteoblast proliferation (Song et al., 2013). Interestingly, the use of the estrogen receptor antagonist, ICI 182, 780, weakened ICA-mediated proliferation and mineralization of osteoblasts, indicating that ICA may function through the ER.

Oxidative stress plays a vital role in decreasing bone density and strength. Low oxygen levels or hypoxia result in an increased generation of reactive oxygen species (ROS) (Miura and Tanno, 2010), which can cause damage to various cellular components, such as proteins, lipids and DNA (Kim and Park, 2003). The antioxidant properties of ICA diminish hypoxia-induced oxidative stress and apoptosis in primary osteoblasts by reducing ROS production and intracellular malondialdehyde (MDA) levels and increasing superoxide dismutase activity. Additionally, it inhibits hypoxia-induced reduction in osteoblast differentiation and mineralization in a dose-dependent manner (Ma et al., 2014). Furthermore, ICA scavenges ROS and maintains osteoblast mitochondrial and primary ciliary homeostasis in diabetes and iron overload-induced OP. ICA also reverses iron overload-induced reductions in the expression of Runx2, alkaline phosphatase and bone-bridging proteins, and significantly prevents bone loss in mice with iron-overloaded (Jing et al., 2019; Liu J. et al., 2022).

The vascular system exerts a pivotal role in bone tissue growth, repair, and remodeling by facilitating nutrient supply and waste removal (Wang and Yeung, 2017). The local blood supply provides essential cells and growth factors for bone regeneration. Notably, specialized cell types within the local vasculature, such as H-type vascular endothelial cells, have been shown to form a complex communication network with various cells in the bone microenvironment (Yang et al., 2026). Studies have demonstrated that H-type vessels can both secrete osteogenic factors and establish physical interactions with bone progenitor cells, thereby directly mediating the “angiogenesis-osteogenesis coupling” process (Kusumbe et al., 2014). ICA has been demonstrated to promote osteogenesis and angiogenesis. In porcine aortic endothelial cells, ICA upregulates the expression of endothelial nitric oxide synthase (eNOS) by activating the EGF-EGFR pathway, thereby modulating endothelial cell function (Liu et al., 2011). Cheng et al. reported that ICA prevents bone loss in ovariectomized rats through activating angiogenesis and inhibiting inflammatory responses (Cheng et al., 2014). Additionally, ICA has been widely utilized in bone defect repair, drug-eluting scaffold development, and biomimetic scaffold fabrication. Zhao et al. constructed a biomimetic scaffold incorporating in situ-encapsulated PLGA@icariin microspheres, which regulates the immune microenvironment and activates the vascular-bone regeneration cascade via the STAT3 signaling pathway (Zhao et al., 2025). Collectively, these studies highlight the potential of ICA as a therapeutic agent with vascular endothelial growth factor (VEGF)-like activity.

In addition to those mentioned above, there are many signaling pathways involved in the treatment of OP. Huang et al. found that ICA could alleviate glucocorticoid (GC)-induced OP by regulating the balance of the EphB4/Ephrin-B2 axis, thereby promoting OP recovery (Huang et al., 2020). In addition, the gut microbiota-bone axis has been recognized as a key mediator of bone homeostasis (Ding et al., 2020). ICA has been shown to prevent bone loss by modulating gut microbiota (GM) and regulating metabolite changes. Potential mechanisms include GM-regulated serum biomarkers RANK, RANKL, OPG and tartrate-resistant acid phosphatase (TRACP), and changes in fecal metabolites such as bile acids, amino acids and fatty acids (Wang et al., 2022c).

### 3.2 Osteoarthritis

Cartilage is an avascular tissue and the absence of blood vessels is considered a key feature of permanent cartilage homeostasis. The intact cartilage and its microenvironment provide balanced support for joint stability (Goldring and Goldring, 2010). Osteoarthritis (OA) is a chronic bone and joint disease characterized by degeneration and destruction of articular cartilage and osteophytes in weight-bearing areas (Zhu et al., 2022). Primary clinical symptoms include pain, swelling and deformation of the knee joint (Martel-Pelletier et al., 2016). Throughout the progression of OA, pathologies affecting the cartilage include oxidative stress, inflammation, apoptosis, cartilage matrix loss and autophagy.

Cellular inflammation and ECM degradation are increasingly recognized as key drivers of cartilage damage in OA. Chondrocytes respond to inflammatory factors in joint tissues and produce proinflammatory factors (Goldring and Marcu, 2009). Interleukin-1β (IL-1β) and tumor necrosis factor-α (TNF-α), catabolic cytokines involved in ECM degradation, promote protease production in chondrocytes, leading to cartilage joint destruction (Chen et al., 2015). A previous study showed that ICA can protect chondrocytes from inflammatory damage by inhibiting the activation of the NF-κB/HIF-2α signaling pathway in a mouse model of bone defect (Wang P. et al., 2020). ICA reduces the expressions of HIF-2α, MMP9 and ADMTS5, while promoting those of chondrocyte-specific markers SOX9, AGG and Col2α, in turn inhibiting cartilage degradation and increasing chondrocyte viability. NO and ROS are key in the progression of OA and can interact and derive a series of highly oxidized free radicals that can lead to cell damage and apoptosis (Ansari et al., 2020; Jiang et al., 2023). Zuo et al. reported that Nrf2, a key redox transcription factor, plays an important role in inhibiting oxidative stress and demonstrated that ICA can reduce excessive ROS production and increase the expression of Nrf2 and downstream antioxidant enzymes to achieve cartilage-protection (Zuo et al., 2019).

Inflammation, oxidative stress and cell-death mechanisms (apoptosis, pyroptosis and autophagy) are closely connected. In OA, inflammation, oxidative stress and autophagy are correlated with chondrocyte apoptosis (Hwang and Kim, 2015). Wang et al. found that ICA induces the upregulation of CYTOR in IncRNA to inhibit the apoptosis of chondrocytes (Wang et al., 2021). In contrast to apoptosis, pyroptosis is pro-inflammatory (Wang et al., 2022b). Pyroptosis is primarily mediated by the NLRP3 inflammasome. On one hand, it accelerates the release of pro-inflammatory cytokines and expands the local inflammatory response; on the other hand, the formation of caspase-1 and gasdermin (GSDM) D-N leads to cell swelling and dissolution, which play a cytotoxic role (Wang et al., 2022b). In a lipopolysaccharide (LPS)-induced OA rat model, ICA was shown to reduce LPS-induced pyroptosis and inhibit collagen formation by arresting the NLRP3 inflammasome-mediated caspase-1 signaling pathway, effectively controlling the local inflammatory response (Zu et al., 2019).

**FIGURE 3:**
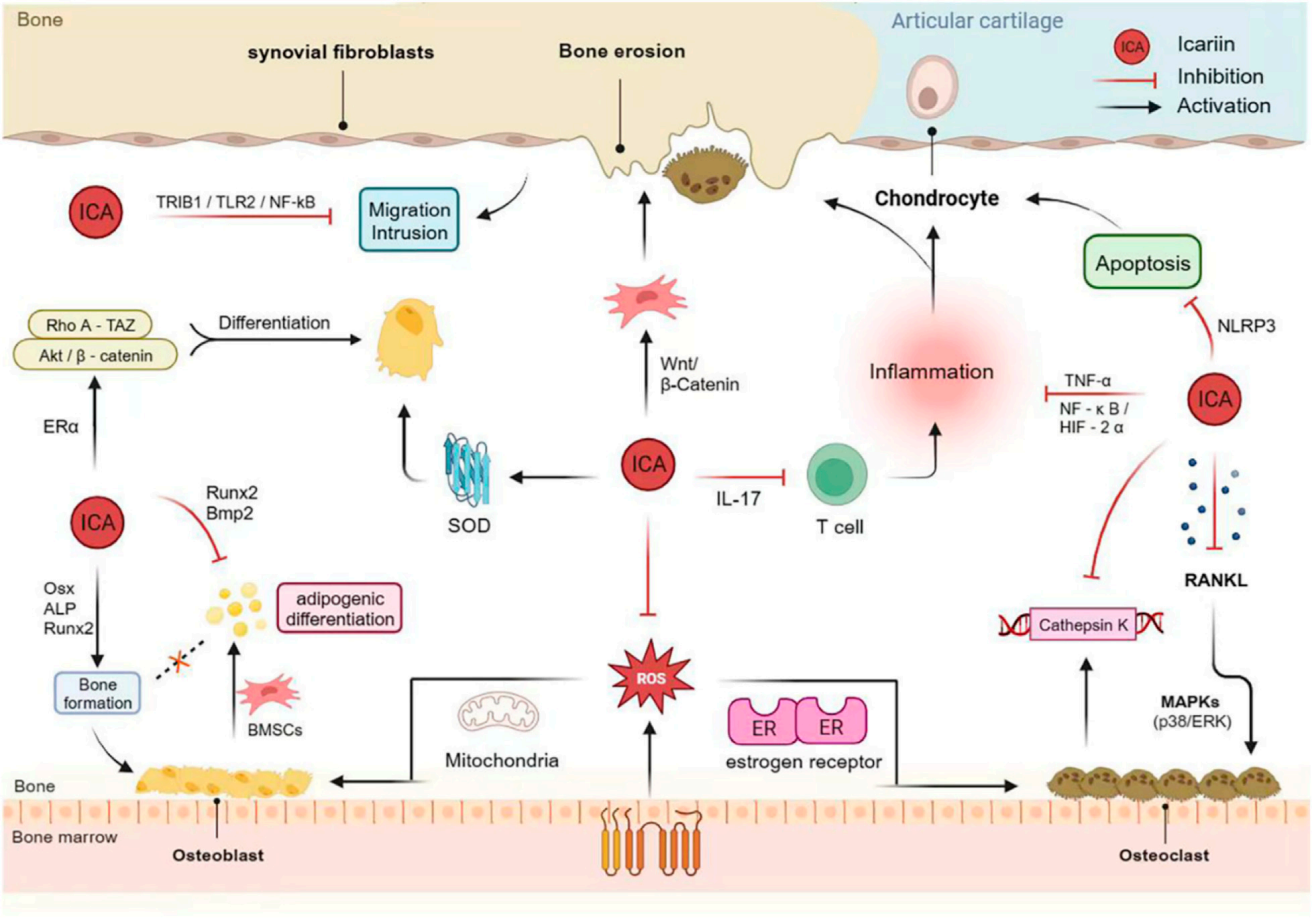
Mechanism of action of Icariin in bone protection and bone damage.

Autophagy is a self-protection mechanism that uses autophagosomes and lysosomes to eliminate damaged organelles and boost cellular adaptation and survival (Li et al., 2019). Consequently, targeted therapy focusing on autophagy is a popular technique in the treatment of OA (Duan et al., 2020). The mammalian target of rapamycin (mTOR) negatively regulates autophagy and is regulated by upstream phosphatidylinositol 3-kinase (PI3K)/AKT and AMP-activated protein kinase (AMPK) signaling (Sun et al., 2020). Tang et al. discovered that ICA activates autophagy and reduces apoptosis in human chondrocytes in a dose-dependent manner. Animal experiments further demonstrated that ICA mediates PI3K/AKT/mTOR signaling, thereby regulating chondrocyte autophagy to alleviate OA (Tang et al., 2021) (Figure 3).

With the rise of regenerative medicine, stem cell and cytokine therapies have become viable options for treating OA (Zhu et al., 2022). Of these, BMMSCs are the most extensively researched and favored source for autologous cell therapy to promote cartilage repair (Ding et al., 2021). Zhu et al. reported that hydrogels loaded with different concentrations of ICA could differentially promote the proliferation and chondrogenic differentiation of BMMSCs through the activation of Wnt/β-catenin signaling *in vitro* (Zhu et al., 2022). Another *in vivo* study showed that intra-articular injections of ICA and BMSCs in female New Zealand rabbits enhanced the BMP2/Smad signaling pathway, which promoted repair of cartilage damage (Jiao et al., 2022). Furthermore, ICA enhances the tolerance of BMMSCs to ischemia and hypoxia, thereby promoting recovery from cartilage damage (Tang et al., 2022). In response to possible growth factor side effects, such as TGF-β stimulating BMMSC differentiation, ICA has been shown to improve the ability of TGF-β to promote BMMSC differentiation into juvenile bone. Interestingly, ICA did not increase the hypertrophic response or fibrotic cartilage formation (Wang et al., 2014).

In addition to the aforementioned factors, iron death is closely linked to OA. Luo and Zhang found that treatment of LPS-induced human synoviocytes with ICA decreased iron content, MDA and other factors associated with iron metabolism, while counteracting the increase in iron content and the imbalance between the oxidative and antioxidant systems induced by the GPX4 inhibitor RSL3 (Luo and Zhang, 2021). Moreover, some medical scientists have turned their attention to exosomes (Exos) (Watanabe et al., 2021); however, studies on the treatment of OA using ICA through modulation of Exos are scarce and deserve more attention in the future.

### 3.3 Rheumatoid arthritis

Rheumatoid arthritis (RA) is an autoimmune inflammatory disease of the joints affecting approximately 1% of the global population. It is characterized by synovial hyperplasia, mononuclear cell infiltration, and the erosion of cartilage and bone (Nagashima et al., 2012). The development of RA is intricate, involving dysfunctional immune cells and synovial fibroblasts, along with unusual expression of multiple autoantibodies and cytokines (McInnes et al., 2016).

Autoimmune diseases often result in inflammatory responses and subsequent tissue damage owing to humoral and cellular immune-mediated immune complex deposition and cytokine production (Khan et al., 2022). The STAT family of signal transducers and activators regulates cell proliferation, apoptosis, angiogenesis and immune response (Shahmarvand et al., 2018). Research has demonstrated that ICA alleviates rheumatoid inflammation and joint destruction by inhibiting the expression of osteoclast markers, such as β3 integrin, cathepsin K and MMP-9. In addition, it reduces the number of Th17 cells and inhibits STAT3 activation-mediated IL-17 production (Chi et al., 2014). Furthermore, it markedly increases mRNA and protein expression of Fas and the activities of caspase8 and caspase3 in CD4 + lymphocytes and decreases mRNA and protein expression of B-cell lymphoma-2 (Wang H. et al., 2017). Previous studies have shown that microRNAs (miRNAs) play an important role in the pathogenesis of RA, and miR-223-3p has been suggested as one of the possible biomarkers of RA (Ogando et al., 2016). ICA inhibits proliferation and inflammatory cytokine secretion by modulating miR-223-33p/NLRP3 signaling, which promotes apoptosis (Wu et al., 2020). Thus, ICA treats RA by modulating immune responses and inflammatory factors.

The hyperproliferation of fibroblast-like synoviocytes (FLS) and synovial angiogenesis are typical pathological features of RA (Lefèvre et al., 2009). Therefore, the inhibition of FLS proliferation is one way to treat RA. p21 is a cell cycle protein-dependent kinase inhibitor that regulates cell cycle progression in response to various stimuli (Karimian et al., 2016). Pu et al. found that ICA upregulates the expression of p21 and downregulates the expression of cytokinins B and E, suggesting that ICA inhibits cell proliferation by interfering with the cell cycle progression of FLS (Pu et al., 2021). ICA also inhibits TNF-α-induced inflammatory response and RA-FLS by regulating the TRIB1/TLR2/NF-κB pathway (Wu et al., 2022). In addition, ICA inhibits iron death by activating the Xc-/GPX4 axis, thereby protecting synovial cells from death (Luo and Zhang, 2021). However, increased invasion and migration of FLS due to inflammatory stimulation of the synovium in patients with RA also accelerates the destruction of articular cartilage and bone (Bottini and Firestein, 2013). Moreover, ICA considerably inhibits the migratory ability of FLS but has a lesser effect on their invasive ability (Pu et al., 2021).

Cathepsin K is expressed in osteoclasts and synovial fibroblasts (Salminen-Mankonen et al., 2007). Overexpression of cathepsin K in mice leads to synovial inflammation and increased cartilage and bone destruction with age, which is a characteristic of RA (Maffia et al., 2004). ICA inhibits the collagenase activity of cathepsin K, resulting in a reduction in clinical inflammation, fibrin deposition, synovial inflammation and cartilage degradation by 11%–25% (Sun et al., 2013).

### 3.4 Degenerative disc disease

Low back pain (LBP) is a common orthopedic condition worldwide that can result in disability and affect daily life. Intervertebral disc degeneration (IVDD) is the primary cause of LBP (Vergroesen et al., 2015). The intervertebral disc (IVD) comprises of the upper and lower cartilaginous endplates (CEPs), nucleus pulposus (NP) and outer annulus fibrosus (Stergar et al., 2019). Despite ongoing research, the pathogenesis of IVDD remains poorly understood and involves a multitude of effector molecules and complex signaling networks (Risbud and Shapiro, 2014). Current treatment options for IVDD include pain management using therapeutic agents and surgery. However, these methods do not achieved the desired therapeutic effects (Sharifi et al., 2015).

Degeneration and calcification of the CEPs can significantly affect the biomechanics and nutrient supply of the IVD, which is a crucial factor in the onset and progression of IVDD (Ashinsky et al., 2020). The CEPs are a layer of hydrated cartilaginous tissue, with chondrocytes as the exclusive cell type (Wang et al., 2016). Various risk factors, including mechanical overload, injury, instability, chemical or genetic disorders and smoking may contribute to the inflammation of CEPs and oxidative stress in chondrocytes, resulting in reduced cell viability and CEP degeneration (Vergroesen et al., 2015; Dowdell et al., 2017). Furthermore, oxidative stress may cause mitochondrial dysfunction and morphological disruption, activating the mitochondrial apoptotic pathway and leading to apoptosis of endplate chondrocytes (He R. et al., 2018). Mitochondrial autophagy is crucial for maintaining mitochondrial homeostasis as it removes damaged mitochondria from the cell and decreases mitochondrial ROS production (Song et al., 2021). In addition to mitochondria-dependent apoptosis, iron death, which is characterized by mitochondrial shrinkage, enhanced mitochondrial membrane density, lipid peroxidation and the involvement of a unique set of genes, decreases cell viability in the NP and is thought to be involved in IVDD (Yang et al., 2021).

Recent mouse experiments have shown (Shao et al., 2022) that the anti-inflammatory and antioxidant activities of ICA prevent CEP degeneration and calcification in the pathology of IVDD. The Nrf-2/HO-1 pathway, activated by ICA, facilitates Parkin-mediated mitosis and inhibits iron-related apoptosis in chondrocytes, which in turn alleviates redox imbalance and mitochondrial dysfunction, ultimately improving cell survival. Hua et al. demonstrated that ICA has a protective effect against H2O2-induced oxidative damage and mitochondria-mediated apoptosis in human NP cells through modulating the Nrf-2 signaling pathway (Hua et al., 2020). Although targeting Nrf2 to regulate lipid peroxidation and iron death is a potential intervention strategy, the specific mechanisms involved require further investigation.

The activation of endogenous stem cells for the restoration and reconstruction of IVDD is considered a superior biological therapy (Huang et al., 2017). ICA enhances the proliferation and growth of neural stem cells in development by regulating cell cycle-related genes and proteins (Fu et al., 2018). Zhang et al. found that ICA can increase the expression of chemotactic cytokines, such as IGF-1, TGF-β, SDF-1 and CCL-5, promoting the migration of intervertebral disc region-derived stem cells to repair degenerated IVDs (Zhang Z. et al., 2022). ICA is a novel and potent long-acting pain reliever that inhibits the overexpression of CINC-1/IL-8; at 100 mg/kg/d, ICA demonstrated analgesic effectiveness similar to that of celecoxib, but with longer-lasting efficacy (Li et al., 2020).

### 3.5 Fracture

Fractures are among the most common types of bone and joint injuries. Most patients with fractures heal naturally rather than by accelerated healing, which is an extremely slow process of bone remodeling (Dimitriou et al., 2011). Bone remodeling includes both bone resorption and formation, which control the remodeling or replacement of bone after an injury, such as a fracture, as well as microinjuries that occur during normal activity (Wei and Cooper, 2023). During bone resorption, active osteoclasts secrete acids and enzymes to break down and resorb the mineralized bone matrix, reducing the amount of bone in the region. During bone formation, osteoblasts continue to differentiate to form new bone tissue (Frost, 2004). Therefore, the search for drugs or interventions that regulate bone remodeling is essential.

Research has shown that ICA is a potential bone-inducing compound for bone tissue engineering (Zhao et al., 2010), and oral administration of ICA promotes bone formation by inhibiting bone resorption and increases peak bone mineral density (BMD) and bone mass (Yang et al., 2014). Bone formation markers (such as osteocalcin (OC) and bone-specific alkaline phosphatase (BAP)) and bone resorption markers, including telopeptides of type 1 collagen (C-terminal: CTX-1 and N-terminal: NTX-1), and serum band 5 tartrate-resistant acid phosphatase (TRACP-5) are low in the blood under normal conditions (Froberg et al., 1999). A study showed that the higher the levels of OC in the bones and blood of rats modeled for femur fractures, the faster the fracture healed (Aydin et al., 2014). Zhang et al. found that ICA upregulated serum levels of OC, BAP, NTX-1 and CTX-1, and activated the BMP-2/Smad5/Runx2 signaling pathway to accelerate fracture healing in relatively young and aged rats (Zhang et al., 2021). *In vivo* experiments showed (Cao et al., 2017) that post-fracture administration of ICA accelerated mineralization and osteogenesis and attenuated BMD loss in osteopenic rats. ICA also promotes MC3T3-E1 cell growth and regulates osteoblast differentiation (Lai et al., 2018). In a rat model of femoral fracture, ICA treatment significantly improved bone mineral density and histopathological outcomes at both the early and late fracture stages (Gürbüz et al., 2019).

A recent study created a titanium dioxide nanotube-polydopamine-ICA (TNT-DP-ICA) drug delivery platform and evaluated its *in vitro* and *in vivo* biological properties. They found that TNT-DP-ICA showed reduced inflammatory response *in vitro*, and after 1 month and 3 months of implantation in fracture rats, the fibrous capsule around the implant decreased, the thickness of newly formed bone tissue increased, and the osseointegration process was significantly improved (Negrescu et al., 2022). Huang et al. designed an antibiotic release system comprising ICA, vancomycin (VA) and calcium phosphate cement to treat infectious bone disease in patients with open fractures (Huang et al., 2013). They discovered that combining ICA with VA could overcome the negative effect of bacteria on bone healing and achieve adequate local drug concentrations. Although further studies are necessary to confirm the therapeutic effectiveness of this strategy for infectious bone diseases, this discovery suggests the potential use of ICA in combination with antibiotics to treat this condition.

### 3.6 Other diseases

In addition to the five aforementioned diseases, ICA exerts therapeutic effects in other bone and joint disorders. One study investigated the role of ICA in a rat model of monosodium urate (MSU)-induced gouty arthritis. MSU increased the degree of ankle swelling, promoted inflammatory cell infiltration, and elevated cytokine levels in rat synovial tissues, all of which were alleviated by ICA (Cao, 2021). Ankylosing spondylitis (AS) is an inflammatory rheumatic disease characterized by spinal inflammation and new bone formation (Kim and Lee, 2023). ICA was found to induce apoptosis of CD4^+^ T cells in patients with AS, thereby exerting anti-rheumatic activity (Wang H. et al., 2017). Additionally, ICA inhibits the expression of fibroblast-specific transcription factors Cbfa1 and Osx via the cytokine BMP-2, thus suppressing fibroblast ossification (Jia et al., 2014). Emerging evidence indicates that ICA may regulate bone metabolism and remodel the tumor microenvironment. Osteosarcoma (OS) is the most common primary skeletal malignancy. Ren et al. demonstrated that ICA inhibits the Wnt/β-catenin signaling pathway and downregulates the expression of downstream phosphorylated p-GSK3β, β-catenin, c-Myc, and cyclin D1, thereby suppressing OS cell proliferation and promoting apoptosis (Ren et al., 2018). ICA also enhances OS sensitivity to the antitumor antibiotic Adriamycin by downregulating multidrug resistance protein 1 (MDR1) and the PI3K/AKT pathways, which may provide novel insights into OS chemotherapy (Wang et al., 2015). Moreover, ICA has been identified as a promising immunomodulator for metastatic prostate cancer (PCa) bone metastasis, as it significantly reduces PCa metastasis-induced bone destruction *in vivo* by inhibiting osteoclastogenesis through downregulation of the TAM/CCL5 pathway (Chen et al., 2023). Recent advances in novel drug delivery systems have highlighted the potential of ICA-loaded biomaterials in bone tissue engineering due to their osteogenic properties. Liu et al. investigated the effect of ICA on the inhibition of peri-implantitis-associated bacteria and developed a calcium phosphate cement composite loaded with ICA-gelatin microspheres, which not only promoted osteoinductivity and bone formation, but also alleviated inflammation (Liu N. et al., 2022). Avascular necrosis of the femoral head is a common hip disease (Ahmed et al., 2023); 3D-printed porous Ti6Al4V reconstruction rods loaded with ICA can effectively promote osteogenesis and neovascularization and interfere with further development of the necrosis (Lei et al., 2023).

## 4 Current status of clinical studies

The efficacy of ICA in bone and joint disease has been demonstrated in numerous clinical trials (Table 1). ICA has been shown to reduce the incidence of osteoporosis in postmenopausal women. The results of a 24-month randomized, double-blind, placebo-controlled clinical trial demonstrated that phytoestrogenic flavonoids (ICA, genistein and daidzein) derived from Epimedium exerted a beneficial effect on preventing bone loss in late postmenopausal women without inducing significant endometrial hyperplasia (Zhang et al., 2007). In particular, long-term administration (up to 12–24 months) of ICA products led to a time-dependent improvement in lumbar spine and femoral neck BMD. However, the effect of ICA on maintaining BMD was relatively modest (Zhang et al., 2007). In another randomized, double-blind, controlled clinical trial of OP patients (360 cases in the Epimedium total flavonoid capsule group and 120 cases in the Gusongbao capsule group), the overall efficacy rates of the main symptoms in the Epimedium total flavonoid capsule group and the control group were 90.83% and 75.00%, respectively, and the rates of BMD improvement were 47.38% and 34.23%. This suggests that Epimedium Total Flavonoid Capsule may increase BMD and improve major symptoms in OP patients (Min et al., 2013). The reported adverse events included rash, constipation, diarrhea, palpitations, tinnitus and gastrointestinal dysfunction, with an incidence of 6.67% (Min et al., 2013). Although ICA has been evaluated in rodent models of bone loss, to date, no studies have reported its efficacy in non-rodent models (Wang et al., 2018). Therefore, further large-scale clinical trials and novel drug delivery systems are needed to explore the efficacy of ICA and its derivatives in bone and joint disorders. Notably, various materials have been developed as scaffolds for ICA delivery, including chitosan/nanohydroxyapatite, porous PHBV (poly(3-hydroxybutyrate-co-3-hydroxyvalerate)) and gelatin/hyaluronic acid composite microspheres (Fan et al., 2012; Xia et al., 2013; Yan et al., 2016), though additional investigations are still required.

**TABLE 1:** Current clinical studies and results of ICA.

Product name	Refs	Research type	Experimental design	Evaluating indicator	Clinical outcome
Epimedium prenylflavonoids(EP)	Yong et al. (2021)	Randomized, double-blind, placebo-controlled clinical trial	58 healthy postmenopausal women were randomly assigned to the EP extract group (740 mg/day) and the placebo group for a 6-week intervention	Safety and pharmacokinetics of EP flavonoid metabolites; Changes in serum bone-specific alkaline phosphatase (BSAP) levels and TRAF6 protein levels in peripheral blood mononuclear cells	Daily administration of EP extract for 6 weeks is safe; The main metabolite in serum is demethylcitric acid; Elevated levels of EP metabolites are associated with elevated levels of the bone synthesis marker BSAP
Epimedium-derived phytoestrogen flavonoids (EPFs)	Zhang et al. (2007)	Randomized double-blind placebo-controlled trial	100 healthy postmenopausal women were randomly assigned to the EPF treatment group (containing 60 mg of ICA, 15 mg of daidzein, and 3 mg of genistein daily) and the control group, both of which supplemented 300 mg of elemental calcium daily for 24 months	Bone mineral density (BMD), urinary deoxypyridinoline (DPD), serum osteocalcin (OC), serum estradiol (E_2_), endometrial thickness	BMD of the lumbar spine and femoral neck in the EPF group remained stable at 12 and 24 months; DPD in urine was significantly reduced in the EPF group, while OC increased slightly; there were no significant changes in serum E_2_ and endometrial thickness in either group, and no serious side effects were observed
Epimedium total flavonoids capsules	Min et al. (2013)	Randomized, double-blind, controlled trial (multicenter)	A total of 480 patients with primary osteoporosis were randomly divided into a treatment group (given Epimedium total flavonoid capsules) and a control group (given Gusongbao capsules), and the effects of treatment before and after treatment were compared between the two groups	Improvement in the main symptoms of OP, bone mass changes (BMD), and overall efficacy rate	The treatment group showed higher overall therapeutic efficacy for primary symptoms, overall bone mass efficacy, and BMD than the control group
Dietary supplement GJ 191 (composed of extracts of epimedium, dioscorea, and salvia)	Li et al. (2025)	Randomized, double-blind, placebo-controlled study	72 adults with mild to moderate knee osteoarthritis and mild to moderate knee pain were randomly assigned to the GJ 191 group and the placebo group for a 12-week intervention	Knee Outcome Questionnaire (KOOS), Visual Analogue Scale (VAS) for pain, knee range of motion, serum C-reactive protein, and stiffness score	The GJ 191 group showed significant improvement in pain VAS assessments at 6 weeks and 12 weeks; GJ 191 supplementation was safe and well tolerated
Herbal adjuvant formula (containing kidney-tonifying herbs and the plant estrogen epimedium)	Deng et al. (2012)	Multicenter follow-up study	194 postmenopausal women were randomly assigned to the treatment group (10 g/day of herbal formula) and the placebo group, both of which received 600 mg of calcium and 400 IU of vitamin D daily for 5 years	Distal radius BMD, potential adverse events, fracture incidence	BMD significantly increased in the treatment group; the incidence of fractures in the treatment group was 2.4 times lower than that in the control group; no significant adverse events were observed in either group

## 5 Toxic side effects

Epimedium, as a Chinese herbal medicine, has been insufficiently studied regarding its systemic toxicity and adverse reactions. Sui et al. evaluated the safety profile of Epimedium aqueous extract through experiments including the mouse bone marrow micronucleus test, the Ames test, and the TK gene mutation test. Results showed that Epimedium is non-mutagenic, with an LD50 exceeding 80 g/kg; its IC50 values in Chinese hamster ovary cells and lung cells were 55.4 and 19.53 mg/mL, respectively, and all toxicity tests yielded negative results (Sui et al., 2006). Recently, a Chinese herbal formula containing 70% Epimedium was reported to cause acute liver failure: two female osteoporosis patients exhibited significant elevations in alanine aminotransferase and aspartate aminotransferase after several months of administration (Pei and Cao, 2009; Zheng et al., 2014). Cheng et al. also found that vomiting, nausea, and decreased locomotion were observed in mice after 3 days of Epimedium administration (Cheng and Cai, 2000). Although epimedium is associated with adverse reactions, the toxic side effects of ICA are negligible. Studies have shown that when NIH mice were gavaged with Epimedium total flavonoids at 36 g/kg/day for 7 days (1440 times the standard human dose), no mortality was observed (Dongmei, 2007). In a normal Wistar rat model, long-term toxicity of Epimedium total flavonoids was evaluated via gavage of the extract at 410 g/kg/day for 12 weeks; no significant differences in any analyzed blood parameters or major pathological changes were found in treated rats (Li et al., 2008). In addition, Zhong et al. evaluated the toxicity changes of Epimedium flavonoids in zebrafish and showed that ICA had negligible toxicity to zebrafish embryos (Zhong et al., 2019). However, other studies have found that ICA can cause developmental toxicity by disrupting thyroid development and hormone synthesis, but this is time- and dose-dependent (Wu et al., 2023). One study showed that ICA protected DNA from excessive oxidative stress in a model of AAPH (2,2′-azobis(2-amidinopropane) dihydrochloride)-induced DNA oxidative damage (Zhao et al., 2007). Notably, the concentrations used (10^–5^ to 10^–4^ M) exhibited clear cytotoxicity, suggesting controversy regarding the extent of ICA-mediated DNA protection against oxidative damage in cellular and animal models. Furthermore, ICA induces osteogenic differentiation of human BMMSCs in a dose-dependent manner, with an optimal *in vitro* concentration range of 10^–9^ to 10^–5^ M; concentrations exceeding 10^–5^ M are cytotoxic (Fan et al., 2011). In summary, no known warnings or contraindications for the use of Epimedium species currently exist, and the optimal dosage remains unclear. Even fewer toxicological studies on ICA have been reported, but at least in the context of bone and joint disorders, ICA has not been found to exhibit significant toxic side effects.

In summary, we have explored the therapeutic role of ICA for various bone and joint diseases as much as possible, but the efficacy and safety of ICA in these diseases are still lacking analysis. To be sure, ICA as a potential drug for bone tissue engineering has been shown to treat bone and joint diseases through multiple pathways and targets, and the related clinical symptoms have been improved with clear efficacy. Nevertheless, head-to-head comparisons of ICA’s efficacy and safety across these diseases are scarce, necessitating more in-depth investigations. In addition, given that ICA is frequently used in combination with other herbs and drugs, understanding its potential drug-drug interactions is critical. These interactions involve not only phase I P450 enzyme genes and drug transporters but also phase II metabolic genes (Li et al., 2012; Zhou et al., 2012; Zhu et al., 2012). In humans, the aromatase cytochrome P450 (CYP19A1) catalyzes the production of estrogen from C19 androgens (Simpson et al., 2002). Aromatase activity is a key factor in bone development and mineralization and is essential for estrogen production in bone (Ribot et al., 2006). Phosphodiesterase 5 (PDE5) inhibitors have been shown to increase aromatase expression and estrogen biosynthesis in human adipocytes and ovarian granulosa cells (Li et al., 2017).

Notably, the PDE5 inhibitor sildenafil and ICA analogues promote osteoblast differentiation by activating the cyclic adenosine monophosphate (cAMP)/protein kinase G (PKG)/Src homology 2 domain-containing tyrosine phosphatase 2 (SHP2) pathway, thereby stimulating aromatase expression (Wisanwattana et al., 2021), providing new insights into the role of estrogen biosynthesis in the treatment of OP. In addition, a related study showed that ICA decreased Cyp2e1 enzyme activity in mice (Min et al., 2009) but induced Cyp3a enzyme activity in rats. Recently, ICA has been shown to inhibit UDP-glucuronosyltransferases, particularly Ugt1 family enzymes, *in vitro*. Xu et al. found that ICA had no inhibitory effect on the expression of P450 and Ugts genes *in vivo*, but that ICA induced the expression of Cyp4a14 in the liver of mice and resulted in a slight increase in the expression of Ugt2b1, Ugt2b5 and Ugt2b36 (Xu et al., 2014). These studies exemplify the potential drug-drug interactions of ICA, but the mechanisms involved are still poorly understood, and more high-quality clinical studies of natural herbal medicines with anabolic and anti-catabolic effects are needed in the future.

## 6 Discussion

Epimedium is a medicinal plant used in various herbal formulas and modern proprietary Chinese medicinal products. Recent studies have indicated that ICA is the most abundant constituent of Epimedium and possesses a wide range of pharmacological activities, including anti-osteoporotic, immunomodulatory, antioxidant, osteoprotective and anti-inflammatory effects. Owing to these properties, ICA has been identified as a potentially beneficial compound for bone protection and treatment of bone damage. This study reviewed the molecular mechanisms of ICA in the treatment of bone and joint diseases. Reducing the expression of inflammatory mediators, preventing oxidative stress and apoptosis, inhibiting FLS proliferation and migration, promoting osteoblast differentiation, and decreasing osteoclast activity are important mechanisms for the treatment of bone and joint diseases using ICA (Table 2). The relevant major pathways and transforming factors include RANKL, IL-1β, TNF-α, NF-κB/HIF-2α pathway, PI3K/Akt pathway, Rho A- TAZ pathway, BMP-2/Smad5/Runx2 pathway, ERα, Nrf2, AMPK and STAT3. Research has shown that ICA, a natural drug, has numerous advantages over surgical and nonsurgical interventions, such as having multiple mechanisms and targets, affordability, low toxicity and minimal side effects.

**TABLE 2:** Literature Reports on icariin in the treatment of bone and joint diseases.

Diseases	Refs	Type of assay	Models	Study description
Osteoporosis	Xu et al. (2019a)	*In vitro*	Bone marrow macrophages (BMMs) and RAW264.7 cells	ICA inhibits RANKL-induced osteoclastogenesis by regulating NF-κB and MAPK.
	Zhang et al. (2016)	*In vitro*	Primary osteoblasts isolated from the cranial bones of newborn NIH mice	ICA upregulates the expression of Runx2 and Bmp2 while downregulating the expression of C/EBPa and Pparγ, thereby inhibiting osteoblasts’ lipogenic differentiation
	Ye et al. (2017)	*In vitro*	Rat adipose-derived stem cells (rASCs)	ICA promotes proliferation and osteogenic differentiation of rat adipose stem cells (r ASCs) through the Rho A- TAZ pathway
	Zhao et al. (2019)	*In vivo* *In vitro*	Female Sprague–Dawley (SD) rats/Bone mesenchymal stem cells (BMSCs)from rats	ICA promotes osteoblast proliferation and differentiation by activating the Akt/β-catenin pathway, in part by upregulating ERα expression
	Xu et al. (2019b)	*In vivo*	Mouse pre-osteoblasts MC3T3-E1 and mesenchymal stem cells C3H10T1/2	ICA promotes osteogenic differentiation and bone formation by inhibiting the Notch signaling pathway
	Ma et al. (2014)	*In vitro*	Neonatal rat calvarial osteoblasts (ROBs)	ICA reduces reactive oxygen species production and intracellular malondialdehyde levels, while also increasing superoxide dismutase activity. This helps to alleviate hypoxia-induced oxidative stress and apoptosis in primary osteoblasts
Osteoarthritis	Wang et al. (2020a)	*In vitro*	Mouse chondrocyte (ADTC5)	ICA protects chondrocytes from inflammatory injury by inhibiting the activation of the NF-κ B/HIF-2 α signaling pathway
	Zuo et al. (2019)	*In vitro*	Human chondrocytes-Adult (HC-A)	ICA significantly rescued IL-1β-induced matrix degradation and excessive ROS production and activated the Nrf2/ARE pathway, thereby protecting cartilage
	Zu et al. (2019)	*In vivo* *In vitro*	Monosodium iodoacetate (MIA)-treated Wistar rats/LPS-treated chondrocytes	ICA alleviates OA by inhibiting NLRP3/caspase-1 signaling mediated pyroptosis
	Tang et al. (2021)	*In vivo* *In vitro*	SD rats/Knee joint chondrocytes	ICA Regulates Autophagy in Chondrocytes by Mediating PI3K/AKT/mTOR Signaling
	Zhu et al. (2022)	*In vitro*	Rat BMSCs	ICA promotes proliferation and chondrogenesis of BMSCs through the Wnt/β-catenin signaling pathway
	Jiao et al. (2022)	*In vivo* *In vitro*	Female New Zealand rabbits/Rabbit BMSCs	Administration of ICA and BMSCs enhances the BMP2/Smad signaling pathway for cartilage injury repair
Rheumatoid arthritis	Chi et al. (2014)	*In vivo* *In vitro*	Collagen-Induced Arthritis (CIA) mice/Mouse bone marrow-derived monocyte/macrophage cells	Inhibition of STAT17 activation by ICA reduces Th17 cells and suppresses IL-3 production
	Wei et al. (2016)	*In vivo*	Antigen-induced arthritis rabbit	ICA regulates bone loss in joints by controlling the expression of RANKL and OPG.
	Pu et al. (2021)	*In vitro*	Human fibroblast-like synoviocytes (FLS) and natural killer cells (NK-92)	ICA induces G2/M phase arrest and apoptosis, thereby inhibiting the migration and proliferation of FLSs
	Wu et al. (2022)	*In vivo* *In vitro*	CIA mice/Human FLS	ICA inhibits TNF-α-induced inflammatory response and RA-FLS by regulating the TRIB1/TLR2/NF-κB pathway
	Sun et al. (2013)	*In vivo* *In vitro*	CIA mice/Mouse Cathepsin K	ICA inhibits the collagenase activity of histone K, thereby attenuating fibrin deposition and synovial inflammation
Intervertebral disc degeneration	Shao et al. (2022)	*In vivo* *In vitro*	IVDD mice/Mouse endplate chondrocytes	ICA-activated Nrf-2/HO-1 signaling pathway promotes the Parkin-mediated mitochondrial autophagy process and inhibits the death of iron in chondrocytes
	Hua et al. (2020)	*In vivo* *In vitro*	IVDD rats/Human nucleus pulposus cells	ICA has a protective effect against H2O2-induced oxidative damage and mitochondria-mediated apoptosis in human NP cells through modulating the Nrf-2 signaling pathway
	Fu et al. (2018)	*In vitro*	Rat primary neural stem cells	ICA Regulates Cell Cycle Gene and Protein Expression to Enhance Proliferation and Growth of Growing Neural Stem Cells
	Li et al. (2020)	*In vitro*	Puncture-induced low back pain rats	ICA inhibits CINC-1/IL-8 upregulation to achieve analgesic efficacy
Fractures	Zhang et al. (2021)	*In vivo* *In vitro*	Rats with traumatic fracture of tibia/Rat BMSCs	ICA accelerates fracture healing by activating the BMP-2/Smad5/Runx2 pathway
	Cao et al. (2017)	*In vivo*	Ovariectomized (OVX) rats with tibial fractures	Post-fracture administration of ICA promotes BMD growth and accelerates osteogenesis

However, many challenges remain regarding the application of ICA in bone and joint diseases. Currently, relevant studies are mostly conducted in cellular and animal models and human clinical trials are scarce, suggesting that the clinical translation of ICA is not imminent. According to the biopharmaceutical classification system, ICA is classified as a class IV drug with low solubility, permeability and bioavailability (Dong et al., 2021). These unfavorable physicochemical and pharmacokinetic factors severely limit its clinical application. Therefore, there is an urgent need to improve the bioavailability and clinical efficacy of ICA. ICA promotes bone formation and bone protection by acting on a variety of proteins or genes; thus, it is essential to further investigate its structure and possible direct targets in cells to promote targeted therapy for various bone diseases. In terms of toxicology, there is a lack of robust evidence from *in vivo* or *in vitro* studies on the chronic, acute and long-term toxicity of ICA, making it difficult to predict the safety and adverse events associated with its use in bone and joint disorders. Larger sample sizes are needed for preclinical and clinical systematic investigation of the side effects and efficacy of ICA. Because ICA and its various derivatives have varying degrees of potency in osteogenesis, work remains to be done to determine which compounds have the strongest osteogenic activity, which is challenging at this time because the full details of their mechanisms of action are unknown and comparative studies are lacking.

This article outlines the potential of plant-derived ICA in the treatment of bone and joint disorders; however, it has several limitations. First, although ICA has shown promising results in cellular and animal experiments for treating bone diseases, only a small number of relevant clinical trials have been retrieved, and evidence from many of these trials is lacking to demonstrate the safety and therapeutic potential of the molecule in patients with bone diseases. Second, most studies cited in this review had small sample sizes, which may have introduced variability and limited the generalizability of their findings. Therefore, large-scale randomized controlled trials are needed to address these limitations and strengthen the evidence base. Third, many gaps need to be addressed in the development of novel drug delivery systems for the treatment of bone and joint diseases. It has been reported that ICA-nanocarrier coupling has good results *in vitro*; however, *in vivo* experiments are needed for further validation (Wang Q. et al., 2017; Zhang et al., 2019). Future research needs to focus on the following aspects: (1) in-depth study of the mechanism of ICA in the treatment of orthopedic diseases, especially involving certain hotspots, such as intestinal flora, iron death, Exos and stem cells; (2) optimizing the drug properties, dosage and dosing regimen of ICA to improve its stability and efficacy *in vivo* while minimizing toxicity and side effects; (3) exploring the relationship between ICA and the common therapeutic strategies for bone diseases (drugs and surgery) when used in combination; (4) Further develop novel drug delivery systems such as nanoparticles and liposomes using ICA as a carrier, or combine ICA with other materials, including polymers and hydrogels, to improve its bioavailability and promote clinical application; (5) To identify the direct molecular targets of ICA for the treatment of bone diseases by using cyberpharmacology or experimental validation to enhance its targeted therapeutic effect.

## 7 Conclusion

In recent years, Chinese medicine has demonstrated unique efficacy in the treatment of bone diseases. This paper presents a thorough review of the mechanisms of action of ICA in different types of bone diseases. Existing research provides scientific evidence that ICA, a natural compound, may serve as a promising supplement in the prevention and treatment of various bone and joint diseases, attributed to its diverse pharmacological activities, including anti-inflammatory, antioxidative, osteogenic, anti-osteoclastic, and anti-apoptotic effects. Although ICA has beneficial effects on bone health, few studies have extended beyond animal models, and a large number of laboratory studies and clinical trials are needed to confirm its efficacy. Further research may establish ICA as a promising drug candidate for the treatment of bone and joint diseases.

## Glossary

ICA: Icariin
BMMSCs: Bone marrow mesenchymal stem cells
OP: Osteoporosis
OA: Osteoarthritis
RA: Rheumatoid arthritis
IVDD: Intervertebral disc degeneration
TCM: Traditional Chinese medicine
ECM: Extracellular matrix
RANKL: Receptor activator of nuclear factor-κΒ ligand
Osx: Osterix
Runx2: Runt-related protein 2
LRP5: Lipoprotein receptor-related protein 5
ESR1: Estrogen receptor α
OPG: Osteoprotegerin
NF-κB: Nuclear factor-κB
ALP: Alkaline phosphatase
Ocn: Osteocalcin
ROS: Reactive oxygen species
TRACP: Tartrate-resistant acid phosphatase
IL-1β: Interleukin-1β
TNF-α: Tumor necrosis factor-α
mTOR: Mammalian target of rapamycin
PI3K: Phosphatidylinositol 3-kinase
AMPK: AMP-activated protein kinase
FLS: Fibroblast-like synoviocytes
LBP: Low back pain
CEPs: Cartilaginous endplates
NP: Nucleus pulposus
BMD: Bone mineral density
OC: Osteocalcin
OS: Osteosarcoma
MDR1: Multidrug resistance protein 1
PCa: Prostate cancer
PDE5: Phosphodiesterase 5
